# Should every region use the same gastric cancer scanning and treatment approaches? let’s reconsider: a northeastern turkey example

**DOI:** 10.1186/s12876-016-0539-8

**Published:** 2016-10-04

**Authors:** Albayrak Fatih, Ozturk Yasin, Dursun Hakan, Albayrak Yavuz

**Affiliations:** 1 Faculty of Medicine, Department of Gastroenterology, Ataturk University, Erzurum, Turkey; 2 Department of Internal Medicine, Erzurum Region Education and Research Hospital, Erzurum, Turkey; 3 Department of General Surgery, Erzurum Region Education and Research Hospital, Türkiye Sağlık Bilimleri University, Erzurum, Turkey; 4 Faculty of Medicine, Department of Gastroenterology, Ataturk University, Erzurum, Turkey

**Keywords:** Gastric Cancer, Esophageal Cancer, Gastric Cancer Scanning, Gastric Cancer Epidemiology, Northeastern Turkey

## Abstract

**Background:**

The rate for upper gastrointestinal (UGI) cancers, and especially the rate for gastric cancer, varies from country to country and from region to region within country. In Turkey, the incidence of gastric cancer varies widely among regions due to the diverse cultures and accompanying food habits of the population. This study aimed to determine the endoscopic frequency of esophageal and gastric cancers and the usefulness of alarm symptoms in diagnosing gastric cancer in subjects undergoing endoscopy in northeastern Turkey.

**Methods:**

This retrospective study was based on hospital records, reviewing the records of patients who had underdone esophago-gastro-duodenal (EGD) video endoscopy at two general hospitals in Erzurum. From July 2010 to January 2013, 25,037 patients from Erzurum underwent EGD procedures under either intravenous sedation or local anesthesia. Classifications of UGI cancer, based on location of the tumor, were defined as esophageal, cardia, cardia and fundus or corpus or all of these, corpus, corpus and antrum, and antrum. Metastasis was studied in 659 patients diagnosed with cancer.

**Results:**

Throughout the study, 1,007 biopsy samples were reported as malignant tumors (719 gastric, 276 esophageal, and 12 duodenal cancers). The study considered the gastric cancer population under age 46, which included 99 (13.8 %) patients. Among them, the distribution of UGI cancer by age was as follows: under age 26 years, 9 patients (0.9 %); age 26–35 years, 30 patients (3 %); and age 36–45 years, 60 patients (6 %). Overall, 298 patients (298/1007, 29.6 %) presented with localized disease, and 361 patients (35.9 %) had distant metastases at the time of diagnosis.

**Conclusions:**

We determined that UGI cancer patients are observed more frequently in northeastern Turkey than in western Turkey, Europe, and the USA. We believe that alarm symptoms and endoscopic scanning programs require new, region-specific criteria to diagnose UGI cancers in this region. For the patient groups with these different characteristics, we believe that new scanning, follow-up, and treatment strategies are needed that take into consideration differences in the histopathology of the tumors, their localization, and the patients’ ages.

**Trial registration:**

There is registration number. This study is “retrospective study”. This study is “retrospectively registered”.

## Background

Upper gastrointestinal (UGI) cancers, including esophageal and gastric cancers, are among the most common causes of cancer deaths worldwide [1]. Globally, gastric cancer is the second most common cancer, with an estimated 870,000 new cases yearly [2], and it has a poor prognosis because in most cases it is advanced when discovered. Early detection and treatment are among the most important strategies for preventing and controlling gastric cancer [3].

The rate of UGI cancer, and especially that of gastric cancer, varies from country to country and from region to region within countries. High-risk areas include Eastern Europe, East Asia (China and Japan), and parts of Central and South America. Low-risk areas include North America, North and East Africa, Southern Asia, Australia, and New Zealand [4]. Turkey has a relatively high rate of gastric cancer, but its incidence varies widely among regions due to the diverse cultures and accompanying food habits of the country’s population [5]. Upper UGI cancers are detected more frequently in Erzurum province, in northeastern Turkey, than in other regions of the country [6–9].

With a population of about 800,000, Erzurum, located in the northeastern part of the Anatolian peninsula in Turkey, is one of the first referral centers for neighboring cities; therefore, it serves a population of about 2.5 million, approximately 3% of Turkey’s population [10]. This study aimed to determine the endoscopic frequency of esophageal and gastric cancers and the usefulness of alarm symptoms for diagnosing gastric cancer in subjects undergoing endoscopy in northeastern Turkey.

## Methods

### Setting and design

A retrospective study was carried out in the adult population (age ≥ 18 years), reviewing the hospital records of patients who had undergone esophago-gastro-duodenal (EGD) video endoscopy at 2 general hospitals in Erzurum: Ataturk University Faculty of Medicine Hospital and Erzurum Region Research and Education Hospital, both of which provide tertiary care and which together serve a population of about 2.5 million. Between July 2010 and January 2013, 25,037 patients from Erzurum underwent EGD procedures under either intravenous sedation or local anesthesia. The study reviewed and included all available patient records from July 1, 2010 to January 1, 2013. The study was conducted according to the principles of the 1975 Declaration of Helsinki and was approved by the Ethics Committee of the Erzurum Region Research and Education Hospital (Approval date/number: 05.03.2013/03). The Ethics Committee of the Erzurum Region Research and Education Hospital does not require informed consent for retrospective studies. Informed consent is not obtained in this study and personal identities is not shared in this manuscript.

Classifications of UGI cancer, based on tumor location, were defined as Eo (esophageal), GCa (cardia), GCaF and Co (cardia and fundus or corpus or all of these), GCo (corpus), GCoA (corpus and antrum), and GA (antrum).

The histopathology of the specimens obtained (10–12 specimens/patient) was independently determined by senior pathologists in the 2 hospitals. Biopsies for detecting Helicobacter pylori (H. pylori) were taken from the gastric antrum for rapid urease testing or histology.

### Data collection

A compilation form was designed to collect the necessary information regarding the following items: 1) socio-demographics, including patient name/ID registry, residence, gender, nationality, and age at diagnosis of gastric cancer and 2) the health facility responsible for referring each patients and the main presentation at diagnosis. Then, histopathological reports were reviewed to delineate the main cytopathological features, the subsites affected, and the lesions’ prominent cytological characteristics. After histopathological diagnosis of cancer, some cases were excluded from follow-up because the patients were lost due to continuing their treatments at other centers. Therefore, metastasis could not be determined in 348 of the 1007 cancer cases. Some patients who had undergone endoscopy had attended another center, at which they had also undergone H. pylori testing, so our endoscopy process only took a biopsy from the lesion for histopathological diagnosis. H. pylori was not present in these patients, as determined by pathological, serological, or other methods. Therefore, the present study did not address the information regarding those patients who had undergone H. pylori testing. The number of gastric cancer cases checked for the presence of H. pylori was 512 (512/719, 71.2 %).

### Data management and statistical analysis

Data management was carried out using the Statistical Package for Social Sciences, SPSS 10 (Chicago, IL, USA). Descriptive statistics (means, standard deviations, and frequencies) were used to describe the studied variables. A Chi-square test was used for cross tabulation. Throughout analysis, the level of significance was set at a *p* value < 0.05.

## Results

### Demography

The retrospective identified 25,037 patients who had undergone EGD procedures. Of these, 1,007 had biopsy samples that were reported as malignant tumors (719 gastric, 276 esophageal, and 12 duodenal cancers). The mean age of the patients at the time of diagnosis was 63.0 ± 13.0 years (63.2 ± 13.8 for females and 62.9 ± 12.3 for males), with the majority of patients being older than 45 years (*n* = 908; 90.2 %). The data revealed a general increase in gastric tumor incidence in both genders and in all age groups. Tables 1 and 2 show the incidence of gastric tumors in the various age groups in females and males, respectively. Cancers were significantly higher in men (*p* < 0.05). The youngest and oldest patients with gastric cancer were 21 and 88. There was a large predominance of newly diagnosed cases in males (*n* = 580; 57.6 %). In women, patients were older at diagnosis than men (63.2 ± 13.8 vs. 62.9 ± 12.3) but no significant difference was noted between the genders (*p* > 0.05). The most common patient age group at diagnosis was 56–65 years (33.7 %). Of those gastric cancer patients under age 46, which was 99 (9.8 %) patients, the distribution of UGI cancer within age groups was as follows: under age 26, 9 patients (0.9 %); age 26–35, 30 patients (3 %); and age 36–45, 60 patients (6 %). It is notable that 9.8 % of the patients were younger than 45; of the total of 276 patients with esophageal cancer, 15 (5.4 %) were younger than 45.

**Table 1:** Incidence of upper gastric cancer in different age groups in males

		Site						
		Eo	GCaF and Co	GCo	GCoA	GA	D	Total
Age group								
Under 45	n	5	14	8	14	6	1	48
	% wag	10.4 %	29.2 %	16.7 %	29.2 %	12.5 %	2.1 %	100 %
	% ws	3.1 %	7.9 %	8.8 %	20.0 %	8.0 %	16.7 %	8.3 %
	% T	0.9 %	2.4 %	1.4 %	2.4 %	1.0 %	0.2 %	8.3 %
46–55	n	23	22	0	24	6	1	76
	% wag	30.3 %	28.9 %	0 %	31.6 %	7.9 %	1.3 %	100 %
	% ws	14.4 %	12.4 %	0 %	34.3 %	8.0 %	16.7 %	13.1 %
	% T	4.0 %	3.8 %	0 %	4.1 %	1.0 %	0.2 %	13.1 %
56–65	n	76	61	47	4	25	2	215
	% wag	35.3 %	28.4 %	21.9 %	1.9 %	11.6 %	0.9 %	100 %
	% ws	47.5 %	34.3 %	51.6 %	5.7 %	33.3 %	33.3 %	37.1 %
	% T	13.1 %	10.5 %	8.1 %	0.7 %	4.3 %	03 %	37.1 %
66–75	n	41	43	33	12	17	1	147
	% wag	27.9 %	29.3 %	22.4 %	8.2 %	11.6 %	0.7 %	100 %
	% ws	25.6 %	24.2	32.3 %	17.1 %	22.7 %	16.7 %	25.3 %
	% T	7.1 %	7.4 %	5.7 %	2.1 %	2.9 %	0.2 %	25.3 %
75 +	n	15	38	3	16	21	1	94
	% wag	16.0 %	4.4 %	3.2 %	17.0 %	22.3 %	1.1 %	100 %
	% ws	9.4 %	21.3 %	3.3 %	22.9 %	28.0 %	16.7 %	16.2 %
	% T	2.6 %	6.6 %	0.5 %	2.8 %	3.6 %	0.2 %	16.2
Total	n	160	178	91	70	75	6	580
	% wag	27.6 %	30.7 %	15.7 %	12.1 %	12.9 %	1.0 %	100 %
	% ws	100 %	100 %	100 %	100 %	100 %	100 %	100 %
	% T	27.6 %	30.7 %	15.7 %	12.1 %	12.9 %	1.0 %	100 %

*Abbreviations*: *wag* within age group, *ws* within site, *T* total, *n* frequency, *Eo* esophageal, *GCaF and Co* Cardia and fundus or corpus or all of them, *GCo* corpus, *GCoA* corpus and antrum, *GA* antrum, *D* duodenum

**Table 2:** Incidence of Upper Gastric Cancer in Different Age Groups in Females

		Site						
		Eo	GCaF and Co	GCo	GCoA	GA	D	Total
Age group								
Under 45	n	10	23	3	12	1	2	51
	% wag	19.6	45.1	5.9	23.5	2.0	3.9	100 %
	% ws	8.6	14.8	9.4	18.5	1.9	33.3	11.9 %
	% T	2.3	5.4	0.7	2.8	0.2	0.5	11.9 %
46–55	n	13	15	8	6	12	1	55
	% wag	23.6	27.3	14.5	10.9	21.8	1.8	100 %
	% ws	11.2	9.7	25.0	9.2	22.6	16.7	12.9 %
	% T	3.0	3.5	1.9	1.4	2.8	0.2	12.9 %
56–65	n	51	43	8	3	18	1	124
	% wag	41.1	34.7	6.5	2.4	14.5	0.8	100 %
	% ws	44.0	27.7	25.0	4.6	34.0	16.7	29.0 %
	% T	11.9	10.1	1.9	0.7	4.2	0.2	29.0 %
66–75	n	32	39	12	27	10	1	121
	% wag	26.4	32.2	9.9	22.3	8.3	0.8	100 %
	% ws	27.6	25.2	37.5	41.5	18.9	16.7	28.3 %
	% T	7.5	9.1	2.8	6.3	2.3	0.2	28.3 %
75 +	n	10	35	1	17	12	1	76
	% wag	13.2	46.1	1.3	22.4	15.8	1.3	100 %
	% ws	8.6	22.6	3.1	26.2	22.6	16.7	17.8 %
	% T	2.3	8.2	0.2	4.0	2.8	0.2	17.8 %
	n	116	155	32	65	53	6	427
	% wag	27.2	36.3	7.5	15.2	12.4	1.4	100 %
	% ws	100	100	100	100	100	100	100 %
	% T	27.2	36.3	7.5	15.2	12.4	1.4	100 %

*wag* with in age group, *ws* with in site, *T* total, *n* frequency, *Eo* esophageal, *GCaF* and *Co* Cardia and fundus or corpus or all of them, *GCo* corpus, *GCoA* corpus and antrum, *GA* antrum, *D* duodenum

Tables 1 and 2 show the frequency of gastric cancer by site among the various age groups in females and males, respectively. The most common location was GCaF and Co (*n* = 333; 33.1 %), followed by Eo (*n* = 276; 27.4 %), GCoA (*n* = 135; 13.4 %), GA (*n* = 128; 12.7 %), GCo (*n* = 123; 12.2 %), and the duodenum (*n* = 12; 1.2 %). When gastric cancer cases were combined, the majority (386/719; 53.7 %) were distal gastric cancers, while a large minority of cases (*n* = 333, 46.3 %) were found on proximal sites of the stomach.

### Morphology

The morphology results confirmed that adenocarcinoma was a common gastric tumor, occurring in 630/719 (87.6 %) patients. More than half of all tumors (54.2 %) were caused by two main types of adenocarcinoma, intestinal and diffuse, and the intestinal type was common carcinoma (32.9 %). As Table 3 shows, gastric tumors were also associated with signet ring cell gastric cancer, occurring in 35/719 (4.9 %) patients, squamous cell carcinoma, occurring in 15/719 (2.1 %) patients, and other tumors, occurring in 39/719 (5.4 %) patients.

**Table 3:** Histological diagnosis of the upper gastrointestinal cancers in patients

Site	Histological type	Frequency / %
Esophageal tumors	Squamous cell carcinoma	207/276 / 75.0 %
	Adenocarcinoma	65/276 / 23.6 %
	Other tumors	4/276 / 1.4 %
Gastric tumors	Adenocarcinoma	630/719 / 87.6 %
	Signet ring cell	35/719 / 4.9 %
	Squamous cell carcinoma	15/719 / 2.1 %
	Other tumors	39/719 / 5.4 %

Squamous cell carcinoma was a common esophageal tumor, occurring in 207/276 (75.0 %) patients. In addition, adenocarcinoma, occurring in 65/276 (23.6 %) patients, and other tumors, which occurred in 4/276 (1.4 %) patients, was also associated with esophageal tumors.

**Fig. 1:**
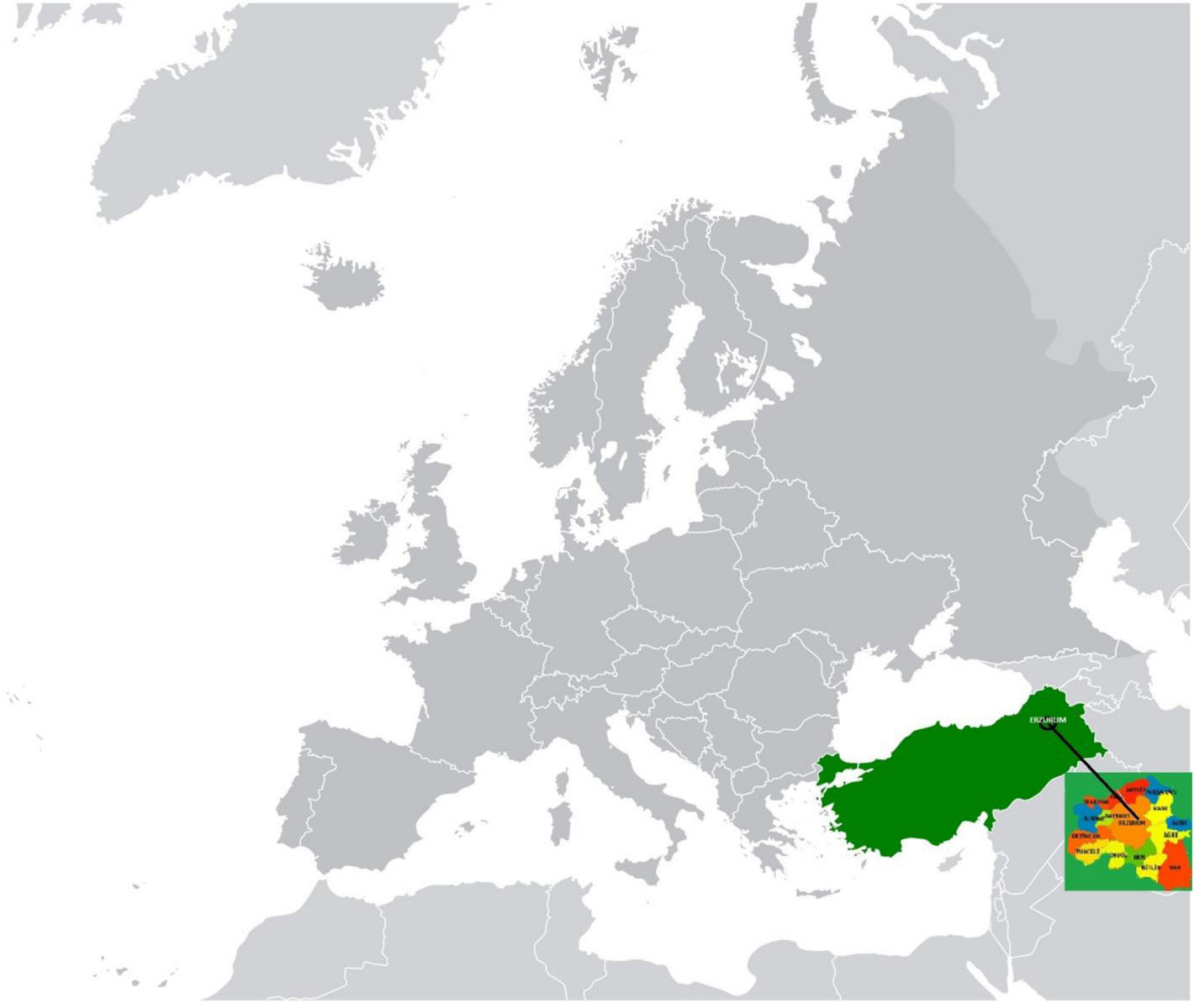
Erzurum is a province of Turkey in the Northeastern Anatolia Region of the country. It is bordered by the provinces of Kars and Ağrı to the east, Muş and Bingöl to the south, Erzincan and Bayburt to the west, Rize and Artvin to the north, and Ardahan to the northeast. These provinces are shown on the Europe map (taken from https://commons.wikimedia.org/wiki/T%C3%BCrkiye#/media/File:Europe_location_Turkey.svg and has been changed by authors).

The geographic distribution was as follows: Erzurum (*n* = 630, 62.6 %), Agrı (*n* = 180, 17.9 %), Kars (*n* = 78, 7.7 %), Igdır (*n* = 37, 3.7 %), Mus (*n* = 36, 3.6 %), and other cities (Van, Ardahan, Bingöl, etc.). This distribution showed that, in addition to Erzurum, cases came most frequently from the northeastern Anatolian region, including Erzurum, Ağrı and Kars (Fig. 1).

Only 659 cases were checked for the presence of metastasis. At the time of diagnosis, 298 patients (298/1007, 29.6 %) presented with localized disease, and 361 patients (35.9 %) had distant metastases, the most common sites for which were lymph nodes (56.1 %), liver (30.7 %), peritonitis carcinomatosa (22.9 %), lungs (11.8 %), neck (4.8 %), and other (5.4 %) (Table 4). The remaining patients (*n* = 348) had various degrees of regional extension or an unknown stage.

**Table 4:** Distribution of the metastasis sites among esophageal and gastric cancer patients (*n* = 659) at presentation

Metastasis site	Esophageal tumors (*n* = 194)	Gastric tumors (*n* = 465)	Total (*n* / %)
Lymph nodes	148 / 22.5 %	219 / 33.2 %	367 / 56.1 %
Liver	49 / 7.4 %	153 / 23.2 %	202 / 30.7 %
Peritonitis carsinamotosa	13 / 2.0 %	138 / 20.9 %	151 / 22.9 %
Lung	31 / 4.7 %	47 / 7.1 %	78 / 11.8 %
Neck	29 / 4.4 %	3 / 0.5 %	32 / 4.8 %
Other	7 / 1.1 %	29 / 4.4 %	36 / 5.4 %

In addition, 512 (512/719, 71.2 %) gastric cancer cases were checked for the presence of H. pylori, and 329 cases (329/512, 65.2 %) were found to be positive, according to either the rapid urease test or histology.

### Discussion

Gastric cancer is the second most common cause of death from cancer worldwide [11]. However, the overall incidence rates for gastric cancer have steadily declined over the past 50 years, particularly in developed countries, leaving the incidence rate of gastric cancer much higher in Asia than in Western and developing countries [12]. The temporal pattern of UGI cancer incidence trends in Western countries is that of decreasing incidence of gastric cancer and a shift in cancer site from the distal to the proximal stomach [13, 14]. This has been noted worldwide in diverse populations [14, 15]. We have no precise information on the epidemiology of UGI cancer in northeastern Turkey, or in Turkey overall. However, many studies have indicated that upper UGI cancers are endemic in Erzurum and its surroundings, and several studies have attempted to uncover the underlying reasons for this [6–9, 16].

Worldwide, algorithms and standard treatment methods are applied to the early diagnosis of gastric cancer. However, the frequency, age group, histology, and anatomic sites of gastric cancer are known to have different characteristics in different geographic regions. This feature prompted the present study, which evaluated the results of UGI endoscopies performed in two tertiary hospitals in Erzurum, in northeastern Turkey, to investigate whether the universal use of current gastric cancer scanning and treatment methods should be re-evaluated based on these results.

Age-standardized incidence rates of gastric cancer are about twice as high in Eastern Asian men as in Eastern Asian women, at 35.4 per 100,000 in males and 13.8 per 100,000 in females [17]. In 2009, the age-standard cancer speed in Turkey was 269.7 per 100,000 in men, and 173.3 per 100,000 in women. The woman-man average cancer incidence is 221.5 per 100,000. In 2009, the age-standard gastric and esophageal cancer speeds in Turkey were 16.2 and 2.6, respectively, per 100,000 in men and 8.1 and 1.3, respectively, per 100,000 in women [18]. In the 25–49 years age group, gastric cancer accounts for 6.7 % of the cancers in Turkey in men and 3.2 % in women [11].

The frequency of stomach cancer varies from region to region in Turkey. A study conducted in southeastern Turkey reported a frequency of esophageal cancer of 0.4 %, and a stomach cancer frequency of 2.1 % in patients who had undergone endoscopy [19]. A similar study reported a frequency of esophageal cancer of 0.3 % and a frequency of stomach cancer of 2.0 % in midwestern Turkey [20]. A study conducted in central Turkey reported an endoscopic frequency of stomach cancer of 4.0 % [21]. A study conducted in northwestern Turkey reported the frequency of esophageal cancer as 0.3 % and the frequency of stomach cancer as 1.8 % [22].

Likewise, the frequency of upper UGI cancers in Turkey varies from region to region. The present study detected 1,007 cancer cases from the 25,037 UGI system endoscopies conducted in the region of the study; 719 of these cases were settled in the gastric region, and 276 were settled in the esophageal region.

Patients were under age 45 in 9.8 % of the gastric cancer cases and in 5.4 % of the esophageal cancer cases. When assessed based on age groups, the gastric cancer rate in patients under 45 in the present study was much higher than the gastric cancer rate in the same age group in Turkey overall. That is to say, gastric cancer occurs at a much higher rate and at a younger age in the eastern and northeastern regions of Turkey than in the other regions of the country. These regional differences may reflect variable regional distributions of environmental or life-style risk factors. Therefore, we suggest that upper UGI endoscopies be recommended for those 45 and older and that further research be conducted into the gastric cancer rates in the eastern and northeastern regions of Turkey.

H. pylori infection is a main factor leading to atrophy, intestinal metaplasia, and cancer development in the stomach [23]. In the present study, 512 patients with gastric cancer had been checked for the presence of H. pylori. We observed that 71.2 % of them were positive for H. pylori. Similarly, in those studies conducted in the same region as the present study, the frequency of H. pylori was reported as 71.5–78 % [24, 25].

Proximal gastric cancer has a worse prognosis than distal gastric cancer because it is deeply invasive, it has a high incidence of lymph node metastasis, and its unclear symptoms result in delayed diagnosis [26]. Unfortunately, the global incidence of proximal gastric cancers has increased compared to distal cancers [12]. The present study, in parallel with the literature, confirmed that proximal gastric cancers occurred at a much higher rate than distal cancers.

In the present study, most of the gastric and esophageal cancer cases were from the cities of Erzurum, Agri, and Kars [27]. In contrast, Turkdogan et al. reported that gastric cancer was endemic in Van and its surroundings [9]. The reason the present study found few cancers in Van and its surroundings is because very few patients from the Van region had undergone endoscopy at the 2 hospitals involved in the present study.

In the region where the present study was conducted, proximal gastric cancers occurred at a much higher rate, as reported previously. However, in contrast to the present study, a multicenter retrospective study of gastric cancers in Turkey found a much higher rate of distal gastric cancers [18]. In other words, gastric cancers do not have the same characteristics in every region of Turkey. The lymphatic drainage and blood buildup differ between proximal and distal gastric cancers; therefore, we believe each geographical region requires new scanning, treatment, and follow-up modalities that are specific to that region [26].

A study involving 796 patients in Istanbul, Turkey reported that 61.9 % of those with gastric cancer had adenocarcinoma (intestinal type) and 31.9 % had signet ring cell (diffused) [28]. In addition, a study by Tural et al. and other studies have reported similar rates [29, 30]. Nevertheless, in the present study, 87.6 % of the patients had adenocarcinoma (intestinal type) and 4.9 % had signet ring cell (diffused) cancer. Different characteristics, such as the settlement of the tumor, are seen in different histological types. This supports our recommendation that treatment and follow-up of various histological types should be specific to each type.

In the present study, lymph nodes were the most frequent site of metastasis. In addition, the study determined that other metastases occurred at a rate similar to those reported in the literature. Remote metastases were reported at lower rates in the literature [31, 32]. However, metastatic cancer rates were higher in the present study. We believe that the most probable reason for this is that the present study researched upper UGI cancers in patients who had underdone endoscopy. Furthermore, for reasons that are not yet known, the cancers in the region of the present study occurred at younger ages and progressed more aggressively.

## Conclusions

We determined that upper UGI cancer patients are observed more frequently in northeastern Turkey than in its western regions, Europe, or the USA. In addition, patients with much higher rates of upper UGI cancer were younger than 45 years. Therefore, we believe that alarm symptoms and endoscopic scanning programs require new, region-specific criteria to diagnose upper UGI cancers in this northeastern Turkey. It is necessary to develop scanning, follow-up, and treatment strategies specific to those patient groups who exhibit these different characteristics, and these strategies must take into consideration differences in the histopathology of the tumors, their localization, and the ages of the patients.

## Abbreviations

EGD: Esophago-gastro-duodenal
Eo: esophageal
GA: antrum
GCa: cardia
GCaF and Co: cardia and fundus or corpus or all of these
GCo: corpus
GCoA: corpus and antrum
UGI: Upper gastrointestinal tract
